# The thalamus in trigeminal neuralgia: structural and metabolic abnormalities, and influence on surgical response

**DOI:** 10.1186/s12883-021-02323-4

**Published:** 2021-07-24

**Authors:** Hayden Danyluk, Jennifer Andrews, Rohit Kesarwani, Peter Seres, Robert Broad, B. Matt Wheatley, Tejas Sankar

**Affiliations:** 1 Division of Surgical Research, Department of Surgery, University of Alberta, 3-002 Li Ka Shing Centre For Research, 11203 - 87 Ave NW, Edmonton, AB T6G 2H5 Canada; 2 Division of Neurosurgery, Department of Surgery, University of Alberta, 2D Department of Surgery, University of Alberta Hospital, 8440-112 Street NW, Edmonton, AB T6G 2B7 Canada; 3 Department of Biomedical Engineering, Faculty of Medicine and Dentistry, University of Alberta, 1098 Research Transition Facility, Edmonton, AB T6G 2V2 Canada

**Keywords:** Thalamus, MRI, Spectroscopy, Volumetry, Microvascular decompression, Percutaneous rhizotomy

## Abstract

**Background:**

Medically-refractory trigeminal neuralgia (TN) can be treated successfully with operative intervention, but a significant proportion of patients are non-responders despite undergoing technically successful surgery. The thalamus is a key component of the trigeminal sensory pathway involved in transmitting facial pain, but the role of the thalamus in TN, and its influence on durability of pain relief after TN surgery, are relatively understudied. We aimed to test the hypothesis that variations in thalamic structure and metabolism are related to surgical non-response in TN.

**Methods:**

We performed a longitudinal, peri-operative neuroimaging study of the thalamus in medically-refractory TN patients undergoing microvascular decompression or percutaneous balloon compression rhizotomy. Patients underwent structural MRI and MR spectroscopy scans pre-operatively and at 1-week following surgery, and were classified as responders or non-responders based on 1-year post-operative pain outcome. Thalamus volume, shape, and metabolite concentration (choline/creatine [Cho/Cr] and N-acetylaspartate/creatine [NAA/Cr]) were evaluated at baseline and 1-week, and compared between responders, non-responders, and healthy controls.

**Results:**

Twenty healthy controls and 23 patients with medically-refractory TN treated surgically (17 responders, 6 non-responders) were included. Pre-operatively, TN patients as a group showed significantly larger thalamus volume contralateral to the side of facial pain. However, vertex-wise shape analysis showed significant contralateral thalamus volume reduction in non-responders compared to responders in an axially-oriented band spanning the outer thalamic circumference (peak p = 0.019). Further, while pre-operative thalamic metabolite concentrations did not differ between responders and non-responders, as early as 1-week after surgery, long-term non-responders showed a distinct decrease in contralateral thalamic Cho/Cr and NAA/Cr, irrespective of surgery type, which was not observed in responders.

**Conclusions:**

Atrophy of the contralateral thalamus is a consistent feature across patients with medically-refractory TN. Regional alterations in preoperative thalamic structure, and very early post-operative metabolic changes in the thalamus, both appear to influence the durability of pain relief after TN surgery.

**Supplementary Information:**

The online version contains supplementary material available at 10.1186/s12883-021-02323-4.

## Background

Trigeminal neuralgia (TN) is a neuropathic facial pain disorder characterized by intermittent, typically unilateral attacks of lancinating pain in the distribution of one or more branches of the trigeminal nerve (cranial nerve V—CNV) [1]. Though occasionally caused by intracranial lesions or multiple sclerosis, TN is typically non-lesional and categorized as: 1) classical TN, associated with neurovascular compression at the root entry zone (REZ) of CNV; or 2) idiopathic TN, without neurovascular compression. More than 50% of TN patients become medically-refractory [2], and are typically offered neurosurgical procedures targeting CNV directly, including microvascular decompression (MVD), percutaneous rhizotomy, or stereotactic radiosurgery. Unfortunately, post-surgical pain recurrence is not uncommon: even with MVD—the most efficacious surgical treatment—early recurrence within two years of surgery occurs in approximately 25% of patients, with a recurrence rate of 4% per year thereafter [3]. 

Given the strong association between TN and neurovascular compression at the CNV REZ, and reports of CNV atrophy in TN [4–6], several studies have used magnetic resonance imaging (MRI) to examine CNV structure more closely, and its relationship to surgical outcome. In particular, diffusion tensor imaging (DTI) has revealed CNV microstructural abnormalities in TN, suggestive of de/dysmyelination or axon loss [5, 7–10]. DTI abnormalities in CNV may predict durability of pain relief after surgery [11, 12], but they are not conclusively superior to the prognostic value of clinical factors such as TN pain character, presence and degree of neurovascular compression, or female sex [13, 14]. Overall, there is still an incomplete understanding of the mechanisms underlying the failure of many TN patients to respond adequately to technically successful surgery. Identifying these mechanisms is important because such patients frequently undergo multiple repeat surgical interventions, with persistently diminished quality of life.

Beyond CNV, the trigeminal sensory system includes second-order neurons in brainstem nuclei receiving afferents from CNV which project—via the ventral trigeminothalamic tract—to third-order neurons residing in the contralateral ventral posteromedial nucleus (VPM) of the thalamus, that in turn project to the somatosensory cortex. Preliminary attempts to examine the brainstem with MRI in TN suggest that microstructural abnormalities exist upstream of CNV as well [15, 16], though DTI in the brainstem is technically challenging and susceptible to error [11]. Despite the well-known structural and functional alterations of the thalamus in chronic pain [17], and its role in the trigeminal sensory system, very few TN studies have directly examined the thalamus or its relationship to treatment outcome [18–21]. Recently, we retrospectively identified pre-operative enlargement of the thalamus contralateral to the painful side of the face in patients with TN [22], though, whether thalamic volume is related to surgical outcome per se remains uncertain.

Accordingly, the motivation for this study was to further elucidate the role of the thalamus in TN. We hypothesized that TN patients exhibit characteristic structural and metabolic abnormalities in the thalamus, and further that these abnormalities are associated with surgical outcome in TN. To test our hypothesis, we used structural MRI and ^1^H-magnetic resonance spectroscopy (MRS) to evaluate the thalamus in TN patients prior to surgery, to identify longitudinal changes in thalamus structure and metabolism occurring in these same patients in the early post-operative period, and to examine the relationship of both to durable post-operative pain relief.

## Methods

### Study participants

This was a prospective, longitudinal study of patients undergoing surgical treatment for TN at a single centre between 2017 and 2020, approved by the Health Research Ethics Board—Health Panel of the University of Alberta. Potential study patients were identified in clinic by any one of three neurosurgeons (authors TS, BMW, RB) and provided informed consent. *Inclusion criteria:* medically-refractory classical or idiopathic TN defined using International Classification of Headache Disorders-III (ICHD-III) criteria[1]; scheduled for surgical treatment by microvascular decompression (MVD) or percutaneous balloon compression rhizotomy (BC). *Exclusion criteria:* confirmed multiple sclerosis or other lesional causes of TN; diagnosed psychiatric illness; history of any prior non-TN neurosurgical procedures. Additionally, 20 healthy control (HC) subjects matched to the TN group in mean age and sex distribution, and without chronic pain or psychiatric conditions, were recruited.

### Data acquisition

TN patients underwent MRI scanning within 1-month prior to surgery (*pre-operative* time-point) and at 5–12 days following surgery (1-week *post-operative* time-point). HC subjects underwent a single MRI scanning session. Scanning was carried out on a 3 T Siemens Prisma Magnetom MRI scanner (Erlangen, Germany) with 64-channel head radiofrequency coil. At every MRI acquisition, participants underwent: 3D T1-weighted structural scan (magnetization-prepared rapid acquisition gradient echo (MPRAGE), field-of-view (FOV) = 250 × 250mm^2^, 208 sagittal slices, 0.85 mm isotropic, repetition time (TR) = 1800 ms, echo time (TE) = 2.37 ms, inversion time (TI) = 900 ms, 8° flip angle, 3:41 min), and 2-dimentional multivoxel MRS scan centred over the VPM thalamus (see below for details) in the coronal-orientation (point-resolved spectroscopy (PRESS), FOV = 160 × 160mm^2^, 1 slice, interpolated voxel size = 5 mm x 5 mm x 10 mm, TR = 1700 ms, TE = 35 ms, 90° flip angle, 1024 repetitions, 2 averages, time = 14:37 min). Participants also completed a pain questionnaire describing pain attack frequency, location, and severity measured with a 0-100 mm Visual Analogue Scale (VAS). All participants were followed for at least 12-months following surgery.

### Clinical characteristics and outcome assessment

The following demographic/clinical data were collected: sex; age; duration of TN since diagnosis; side-of-pain; pre-operative pain severity (measured using VAS); first (virgin) or repeat surgical treatment for TN; surgery type (MVD or BC); and medications. Additionally, we determined neurovascular compression (NVC) severity scores for each patient based on the scoring system of Sindou et al.[23] (0: no neurovascular contact or venous contact alone; 1: arterial contact with no indentation of nerve root; 2: arterial contact with displacement and distortion of nerve root; 3: arterial contact with marked indentation in nerve root). NVC severity scores were derived by a single observer (author TS) by examining routine high resolution clinical pre-operative T2 weighted MRI scans, confirmed (in patients undergoing MVD) by intraoperative findings as indicated in operative reports. Note that, in our practice, patients who are found to have no compressive arterial NVC at the time of MVD also undergo internal neurolysis (IN). Study participants were classified as responders or non-responders as follows: *responders* – 1) documented evidence of immediate and persistent pain relief for at least one year after surgery (Barrow Neurological Institute (BNI) facial pain score IIIa or better) [24]; and 2) no offer of or repeat surgical TN treatment; non-responders – 1) inadequate initial pain relief from surgery or early pain recurrence within one year of surgery; or 2) offered or underwent repeat surgical treatment within one year.

### MRI analysis

#### Automated thalamus volumetry and shape analysis

T1-weighted MPRAGE images were used for thalamus volume and shape assessment. Image orientation depended on the analysis performed: 1) *native orientation* analysis—images remained unflipped, in their native orientation; 2) *ipsilateral orientation* analysis—images from patients with left-sided TN were left–right flipped with FMRIB’s FSL toolbox [25] such that the side-of-pain was on the right side of the image, while images from right-sided TN patients were not flipped. This permitted ipsilateral to contralateral side-of-pain comparisons. FMRIB’s FSL brain tissue segmentation toolbox SIENAX [26] was used to generate brain tissue (grey matter, white matter, cerebrospinal fluid) volumes and an estimate of intracranial volume (v-scaling factor). Thalamus segmentations were derived using FSL-FIRST, part of the FSL toolkit (http://fsl.fmrib.ox.ac.uk/fsl/fslwiki/) [27]. FIRST is a model-based segmentation tool that uses shape- and appearance-based models constructed from manually segmented images [28]. Quality control was performed for each patient by two expert raters (authors HD and JA), who inspected all thalamus segmentations; evidence of mis-segmentation resulted in subject exclusion from volumetric analysis. Shape analysis was performed using the vertex analysis extension of the FSL-FIRST toolbox with the standard recommended parameters in the FSL user guide (https://fsl.fmrib.ox.ac.uk/fsl/fslwiki/FIRST/UserGuide) [28]. Vertex-wise shape analysis was designed to assess between group differences on a per-vertex basis using a multi-variate General Linear Model. Meshes of the thalamus were generated for each subject, and to normalize for inter-individual head size differences, the meshes were reconstructed in MNI space. p < 0.05 was considered statistically significant, corrected for multiple comparisons (family wise error, FWE) using a cluster-wise approach.

#### Metabolic assessment of the thalamus

MRS images were used for metabolic assessment of the thalamus. Native orientation raw RDA image files for every time-point were processed using LCModel version 6.3-1L [29]. Individual ^1^H spectra were generated for every interpolated voxel acquired. For every patient at every time point, an MRS voxel (5 × 5 x 10 mm) was placed bilaterally to encompass the left and right VPM thalamus, with the epicenter of each voxel defined according to the following atlas-based coordinates: x = 13 mm lateral to the mid-commissural point, y = 4 mm anterior to the posterior commissure, and z = 1 mm inferior to the mid-commissural point [30] (Supplementary Figure 1). Absolute concentrations of Choline (Cho), N-Acetylaspartate (NAA), and creatine (Cr) obtained from ^1^H spectra within each target voxel were combined to generate relative intra-voxel concentrations of Cho/Cr and NAA/Cr (using Cr as an internal reference), and subsequently used for all MRS analyses. All ^1^H spectra were visually inspected for quality control.

### Statistical analysis

Within-group left-versus-right, or ipsilateral-versus-contralateral, comparisons of thalamus volume and metabolite concentration were performed using Wilcoxon signed-rank tests. In left-versus-right comparisons, we also calculated inter-hemispheric percent differences in volume and metabolite concentrations, using the following formulas: 1) in left TN patients, %interhemispheric difference = (right – left) / left * 100; 2) in right TN patients, %interhemispheric difference = (left – right) / right * 100. For HC subjects, the selected formula depended on the desired between-group comparison (left-sided TN vs. left-HC, formula #1; right-sided TN vs. right-HC, formula #2). Between-group comparisons were performed using Mann–Whitney tests. Within-patient comparisons of pre-operative versus 1-week post-operative thalamus metabolite concentrations were performed using Wilcoxon signed-rank tests. Clinical characteristics and demographic variables were compared using Mann–Whitney tests, as well as Chi-square or Fisher’s exact test where appropriate. Statistical analyses were carried out with GraphPad Prism version 8 for Mac OS X (GraphPad Software, La Jolla California, USA).

Statistical significance was set at *p* < 0.050 (2-tailed).

## Results

### Study Participants

Twenty-three TN patients and 20 HC were included in this study between 2017 and 2020 (Table 1). All 23 TN patients were included in the volumetric analysis, while only 19 TN patients were included in the metabolite analysis because of inadequate spectral quality (n = 2) or failure to acquire MRS scans (n = 2).

**Table 1:** Comparison of demographic and clinical characteristics between TN patients and healthy controls (HC), as well as within TN patients (responders vs. non-responders)

	**Responders**	**Non-Responders**	***P*-value** **(2-tailed)**	**TN**	**HC**	***P*-value (2-tailed)**
Total #	17	6	-	23	20	-
Sex (Female/Male)	8/9	6/0	0.048*	14/9	11/9	0.70
Age (years)	58.6 $\pm$ 9.7	47.4 $\pm$ 10.3	0.016*	56.3 $\pm$ 10.4	54.9 $\pm$ 9.4	0.65
Duration of TN (years)	4.6 $\pm$ 3.3	8.8 $\pm$ 4.7	0.061	5.3 $\pm$ 3.9	N/A	-
Side of pain (left/right)	6/11	2/4	> 0.99	8/15	N/A	-
Pre-op VAS (mm)	79.9 $\pm$ 24.1	66.8 $\pm$ 38.7	0.50	77.6 $\pm$ 27.3	N/A	-
NVC (yes/no)	14/3	3/3	0.27	17/6	N/A	-
NVC score (0/1/2/3)	3/3/6/5	3/0/2/1	0.64	6/3/8/6	N/A	-
Virgin (yes/no)	14/3	3/3	0.28	17/6	N/A	-
Surgery type (MVD/BC)	13/4	3/3	0.32	16/7	N/A	-
Carbamazepine/ oxcarbazepine (yes/no)	15/2	6/0	> 0.99	21/2	N/A	-
Gabapentin/pregabalin (yes/no)	7/10	5/1	0.16	12/11	N/A	-
Other antiepileptics (yes/no)	2/15	1/5	> 0.99	3/20	N/A	-
Antidepressant (yes/no)	2/15	1/5	> 0.99	3/20	N/A	-
Baclofen (yes/no)	2/15	4/2	0.021*	6/17	N/A	-
Opioid (yes/no)	0/17	1/5	0.26	1/22	N/A	-
Cannabis oil (yes/no)	1/16	1/5	0.46	2/21	N/A	-

Mann Whitney, and Chi-square or Fisher’s-exact tests used where appropriate. Means $\pm$ standard deviations are presented. NVC (yes/no): neurovascular compression; NVC score (0/1/2/3)[23]: degree of neurovascular compression; Virgin (yes/no): first-time surgical treatment for TN; *MVD* microvascular decompression; *BC* balloon compression rhizotomy; other antiepileptics: lamotrigine, topiramate; antidepressant: amitriptyline, duloxetine.

### Clinical characteristics and demographics

*All TN:* Clinical and demographic features of all 23 TN patients and 20 HCs are presented in Table 1. TN and HC (14F/9 M and 11F/9 M, p = 0.70). Average TN duration was 5.3 $\pm$ 3.9 years, with right- groups were well matched in age (56.3 $\pm$ 10.4 years and 54.9 $\pm$ 9.4 years respectively, p = 0.65) and sex distribution sided TN more common than left-sided TN (15R/8L). Neurovascular compression was identified in 17/23 TN patients, and pre-operative VAS was 77.6 $\pm$ 27.3 across the entire TN patient group. This study included virgin surgical procedures for 17/23 TN patients, with MVD most common (16 MVD, 7 BC). All TN patients were on antiepileptic medication at surgery, including carbamazepine/oxcarbazepine (n = 21) and/or gabapentin/pregabalin (n = 12). Three TN patients were also on antidepressant/anxiolytic medication, six were on baclofen, one was taking opioids, and two others were taking cannabis oil.

*By response to surgery*: In total, there were 17 responders to surgery and 6 non-responders. Non-responders were exclusively female (6F/0 M), while responders had a balanced sex distribution (8F/9 M). Non-responders were younger than responders (47.4 $\pm$ 10.3 years and 58.6 $\pm$ 9.7 years respectively, *p* = 0.016), and showed a trend toward longer duration of TN compared to responders (8.8 $\pm$ 4.7 years versus 4.6 $\pm$ 3.3 years respectively, p = 0.061). Neurovascular compression severity score was not statistically different between outcome groups (*p* = 0.64, Fisher’s exact test), nor was frequency of surgery type (*p* = 0.32, Chi-square test). The proportion of patients taking baclofen was higher in non-responders than responders (*p* = 0.021), with no other differences in medication use. Individual TN patient clinical profiles are presented in Table 2.

**Table 2:** Clinical characteristics of individual TN patients

	**Sex**	**Age (years)**	**Side**	**Duration (years)**	**Pre-op VAS (mm)**	**Branch**	**NVC score**	**SX types**	**# prev** **SX**	**Medications**
***Responders:***										
1	M	57.5	R	6	66	3	2	MVD	0	carbamazepine
2	M	49.0	R	1	100	1/2/3	2	BC	3	oxcarbazepine, baclofen
3	M	45.1	L	9	98	1	2	MVD	0	carbamazepine, pregabalin
4	F	58.5	R	11	100	2/3	3	MVD	0	carbamazepine
5	M	63.9	R	8	82	1/2/3	2	MVD	0	carbamazepine
6	M	67.5	R	6	71	2/3	1	BC	1	carbamazepine
7	F	74.1	L	3	81	2	3	MVD	0	oxcarbazepine, pregabalin
8	F	65	R	1	93	3	1	MVD	0	gabapentin
9	F	60.3	L	6	36	2/3	3	MVD	1	gabapentin, amitriptyline
10	F	64.9	L	7	100	2/3	3	MVD	0	carbamazepine, gabapentin
11	F	60.4	L	7	86	2/3	2	MVD	0	carbamazepine, oxcarbazepine
12	F	60.4	R	15	80	2/3	0	BC	0	carbamazepine
13	M	41.8	R	2	89	1/2	2	MVD	0	carbamazepine, gabapentin, topiramate
14	F	68.5	L	1	100	3	1	MVD	0	carbamazepine
15	M	61.5	R	2.5	79	3	0	MVD/IN	0	carbamazepine
16	M	63.3	R	2.5	95	2/3	3	MVD	0	oxcarbazepine, lamotrigine, gabapentin
17	M	40.6	R	1	15	2/3	0	BC	0	carbamazepine, baclofen, duloxetine, cannabis oil
***Non-Responders:***										
18	F	37.3	L	6	63	2/3	0	MVD/IN	0	oxcarbazepine, baclofen
19	F	48.9	L	8	2	1/2/3	2	MVD	0	carbamazepine, gabapentin, baclofen
20	F	56.2	R	13	60	1/2/3	2	BC	1	carbamazepine, gabapentin, lamotrigine, baclofen, hydromorphone
21	F	55.5	R	3	100	1/2/3	0	BC	0	oxcarbazepine, gabapentin
22	F	57.5	R	13	100	1/2/3	0	BC	2	carbamazepine, gabapentin
23	F	36.3	R	3	89	2/3	3	MVD	0	carbamazepine, gabapentin, baclofen, amitriptyline, cannabis oil

*M* male; *F* female; *VAS* visual analogue scale; *Branch* affected trigeminal nerve branches; *NVC* neurovascular compression; *SX* surgical treatment; *MVD* microvascular decompression; *BC* balloon compression rhizotomy; *IN* internal neurolysis

### Thalamus structure

#### Native orientation analysis

**Fig. 1:**
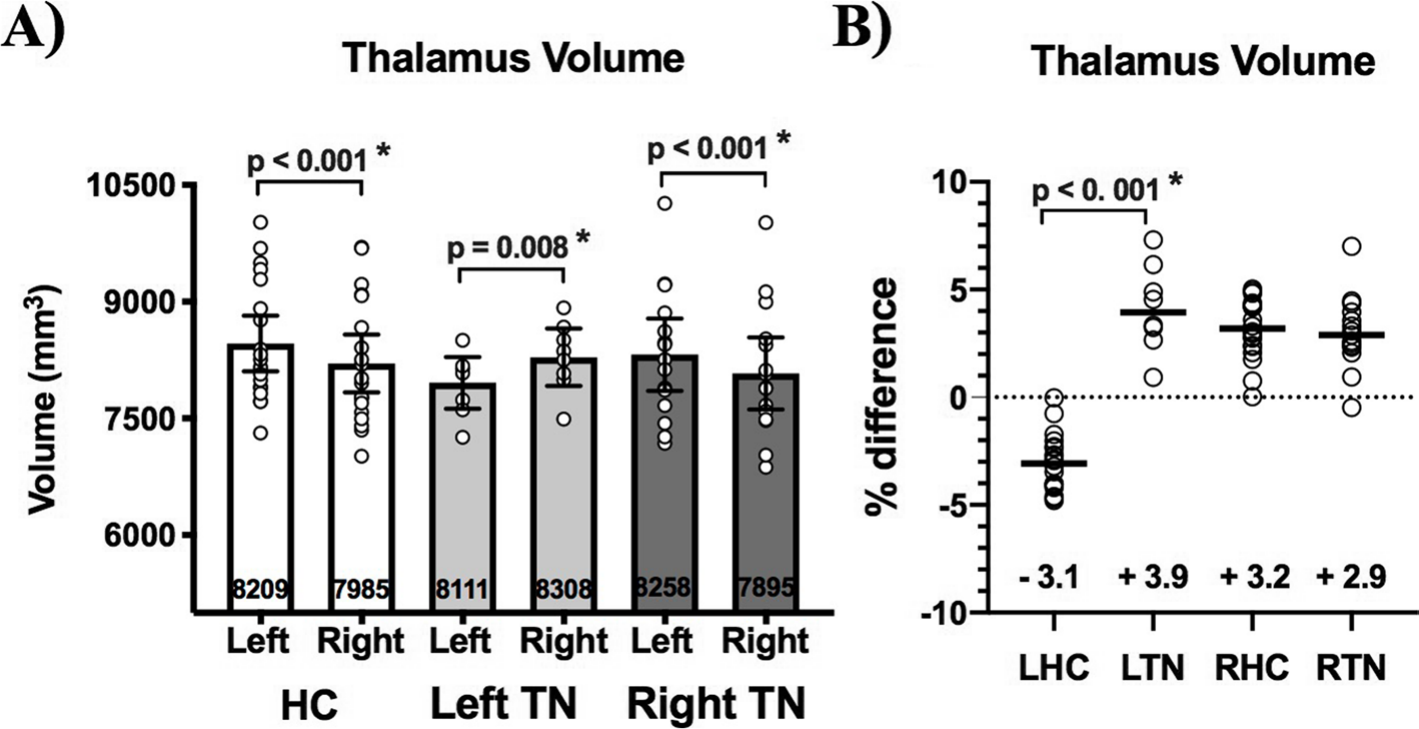
Pre-operative thalamus volume in healthy controls (HC) and TN patients sorted by side-of-pain. **(A)** The left thalamus is larger than the right thalamus (*p* < 0.001) in healthy controls (HC). The contralateral thalamus is larger in TN patients regardless of the side-of-pain (left TN = LTN, right TN = RTN). **(B)** The left vs. right thalamus inter-hemispheric volume % difference differs (*p* < 0.001) between LTN and left-side healthy controls (LHC—see text for definition), while there is no difference in left vs. right thalamus inter-hemispheric % volume percentage difference between RTN and right-side healthy controls (RHC). Mann–Whitney tests were used to perform between-group comparisons, while Wilcoxon sign-rank tests were used to compare thalamus volumes within-groups. Statistical significance (p < 0.05) is indicated with *. Medians with interquartile ranges are displayed

In HCs, the volume of the left thalamus was larger than the right (8209, 7895–9195 IQR and 7985, 7607–8974 IQR respectively; *p* < 0.001). There was no difference between left and right thalamus volume across all TN patients together (8077, 7590–8520 IQR and 8138, 7665–8506 IQR respectively; *p* = 0.88) (data not shown). The volume of the thalamus contralateral to the side-of-pain was larger in TN patients: in left TN patients (LTN) the right thalamus was larger than the left (8308, 8017–8633 IQR and 8111, 7641–8168 IQR respectively; p = 0.008); in right TN patients (RTN) the left thalamus was larger than the right (7895, 7485–8520 IQR and 8258, 7665–8858 IQR respectively; *p* < 0.001) (Fig. 1A). TN was associated with altered interhemispheric asymmetry of thalamic volume: left versus right thalamus interhemispheric %volume difference differed between HC and LTN patients (-3.1, -4.1 to -2.3 IQR; 3.9, 2.8–5.8 IQR; *p* < 0.001), while there was no difference between HC and RTN patients (3.2, 2.4–4.3 IQR; 2.9, 2.3–4.0 IQR; p = 0.54) or between LTN and RTN (3.9, 2.8–5.8 IQR; 2.9, 2.3–4.0 IQR; *p* = 0.13) (Fig. 1B).

#### Ipsilateral orientation analysis

**Fig. 2:**
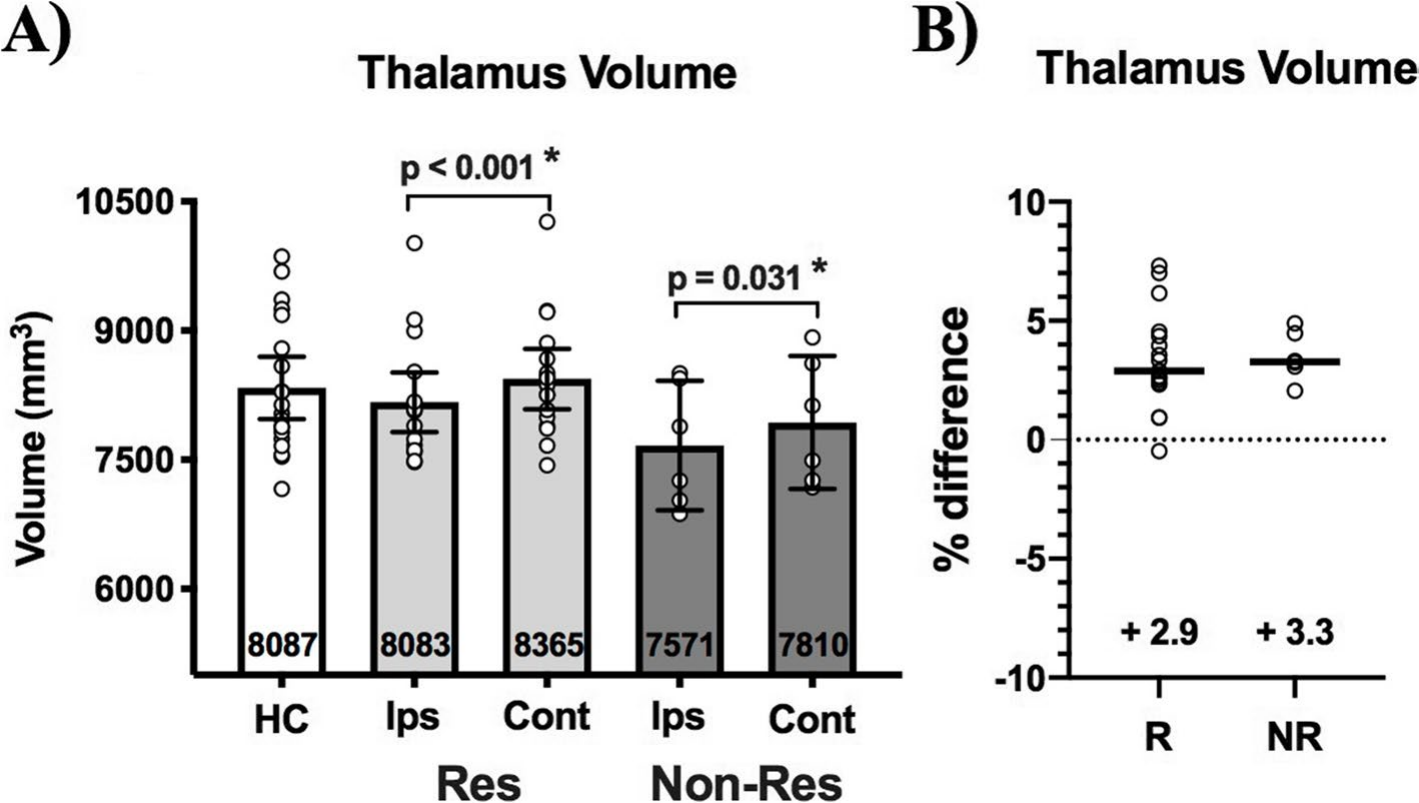
Pre-operative thalamus volume in healthy controls (HC) and TN patients sorted by surgical outcome. There is no difference between HC thalamus volume (average of left and right thalamus) and ipsilateral or contralateral thalamus volume for either responders (Res—light grey bars) or non-responders (Non-Res—dark grey bars). **(A)** The contralateral thalamus is larger than the ipsilateral thalamus in both responders and non-responders. No differences were observed in thalamus volume between responders and non-responders, either ipsilaterally or contralaterally. **(B)** There is also no difference in left vs. right thalamus interhemispheric % volume difference between responders and non-responders. Mann–Whitney tests were used to perform between-group comparisons, while Wilcoxon sign-rank tests were used to compare thalamus volumes within-groups. Statistical significance (*p* < 0.05) is indicated with *. Medians with interquartile ranges are displayed

After flipping the brains of left TN patients, we found no differences between average HC thalamus volume (8087, 7763–9084 IQR) and ipsilateral (ips) or contralateral (cont) thalamus volume of responders (ips: 8083, 7639–8348 IQR, *p* = 0.54; cont: 8365, 7944–8766 IQR, *p* = 0.48) or non-responders (ips: 7571, 6988–8461 IQR, *p* = 0.093; cont: 7810, 7239–8695 IQR, *p* = 0.32). Thalamus volume contralateral to the side-of-pain was larger than ipsilateral to the side-of-pain in both responders (*p* < 0.001) and non-responders (*p* < 0.031) (Fig. 2A), though interhemispheric %volume difference did not differ between outcome groups (R: 2.9, 2.4–4.4 IQR; NR: 3.3, 2.8–4.6 IQR; *p* = 0.71) (Fig. 2B). We did not observe any significant changes in thalamus volume between pre-operative and 1-week post-operative time points in TN patients regardless of side-of-pain or surgical outcome (data not shown).

#### Intracranial volume

Intracranial volume as assessed using the v-scaling factor in FSL’s SIENAX tool did not show any significant differences between any groups across all volumetric comparisons (data not shown).

#### Shape analysis

**Fig. 3:**
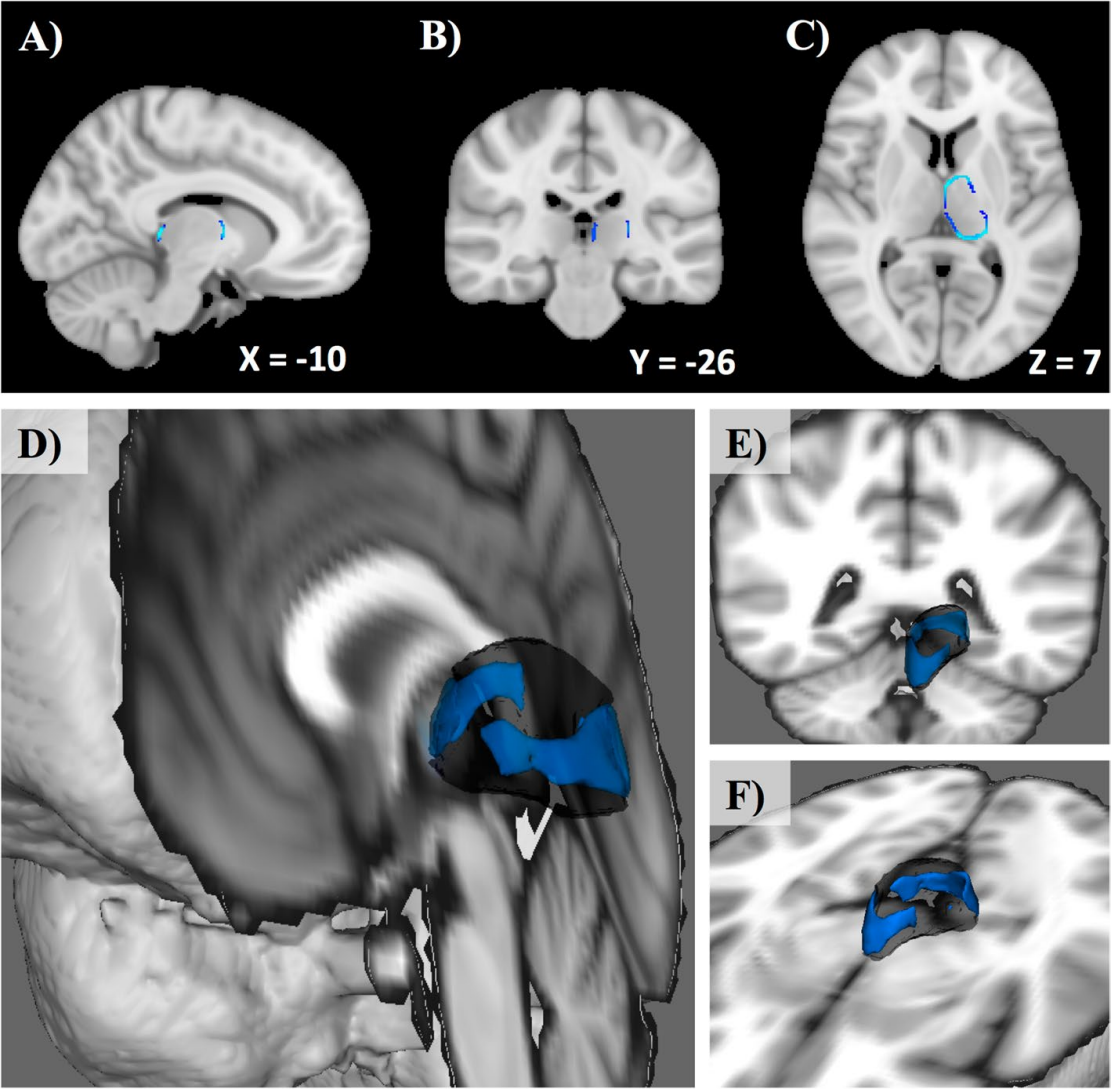
Pre-operative contralateral thalamus shape differences between responders and non-responders to surgical treatment for TN. Results are overlaid on Montreal Neurological Institute (MNI) standard space and are displayed in sagittal- (**A**), coronal- (**B**), and axial-views (**C**). Contralateral thalamus volume loss is observed (blue, p < 0.05) in non-responders compared to responders within an axially-oriented band spanning the outer thalamic circumference made up of two voxel clusters (peak p-value = 0.019), shown in 3-D renderings of the thalamus in **D-F**. MNI coordinates of cross-sectional slice are displayed in figures **A**-**C. **VPM thalamus metabolism:

There were significant vertex-wise shape differences seen between the thalami of responders and non-responders (Fig. 3). Non-responders showed significant contralateral thalamus volume reduction compared to responders in an axially-oriented band spanning the outer thalamic circumference made up of two clusters (peak p = 0.019).

#### Native orientation analysis

**Fig. 4:**
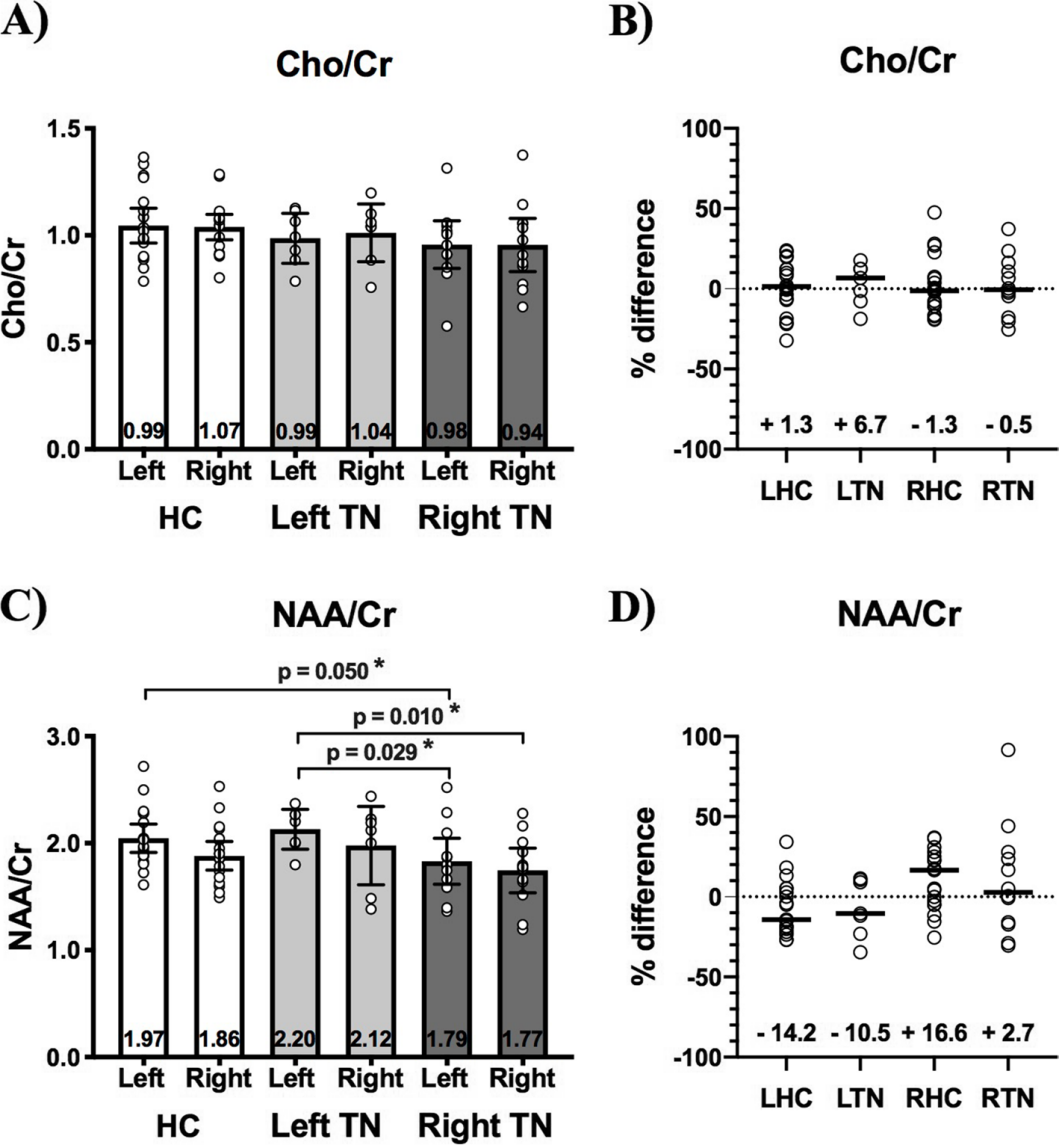
Pre-operative thalamus Cho/Cr and NAA/Cr in healthy controls (HC) and TN patients sorted by side-of-pain. (**A)** There is no difference in Cho/Cr between the left and right thalamus within healthy controls (HC), left-sided TN patients (LTN), or right-sided TN patients (RTN). Additionally, there is no difference in thalamus Cho/Cr between any of these groups. (**C)** Left NAA/Cr is increased in HC compared to RTN (*p* = 0.050). NAA/Cr of the left thalamus is increased in LTN patients compared to both the left (*p* = 0.029) and right (*p* = 0.010) thalamus of RTN patients. Left vs. right interhemispheric % difference for Cho/Cr (**B**) or NAA/Cr (**D**) does not differ between left healthy controls (LHC) and LTN or right healthy controls (RHC) and RTN. Mann–Whitney tests were used to perform between-group comparisons, while Wilcoxon sign-rank tests were used to compare thalamus volumes within-groups. Statistical significance (*p* < 0.05) is indicated with *. Medians with interquartile ranges are displayed

There was no pre-operative difference in Cho/Cr between left and right VPM thalamus in HC, RTN, or LTN patients. Overall, across HC and TN patients, there was no difference in mean Cho/Cr (Fig. 4A), nor any difference in %inter-hemispheric Cho/Cr difference between LHC and LTN patients (Fig. 4B).

In HCs, there was a trend towards left greater than right VPM thalalmus NAA/Cr (1.97, 1.88–2.28 IQR and 1.86, 1.63–2.04 IQR respectively; *p* = 0.060). There was no difference between left and right NAA/Cr in LTN or RTN patients. Left thalamus NAA/Cr was higher in HC compared to RTN patients (*p* = 0.050). Additionally, NAA/Cr of the left (ipsilateral) VPM thalamus in LTN patients was greater than the left (contralateral) and right (ipsilateral) VPM thalamus in RTN patients (*p* = 0.029 and *p* = 0.010 respectively). There were no other between-group differences (Fig. 4C, 4D).

#### Ipsilateral orientation analysis

**Fig. 5:**
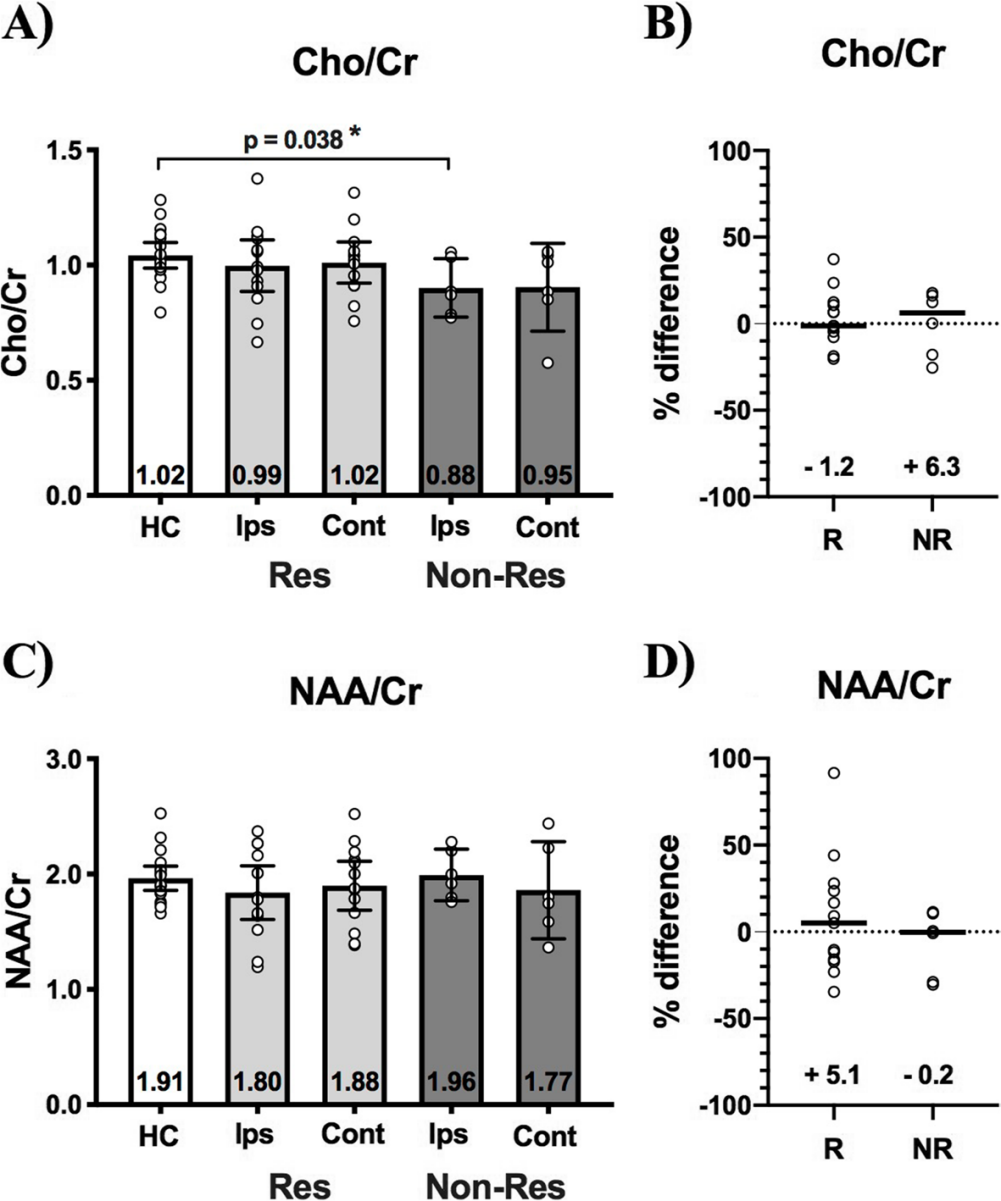
Pre-operative thalamus Cho/Cr and NAA/Cr in healthy controls (HC) and TN patients sorted by surgical outcome. **(A)** Compared to healthy controls (HC), Cho/Cr in non-responders (Non-Res—dark grey bars) is reduced ipsilateral (Ips) to the side-of-pain (*p* = 0.038). There are no between-side differences in thalamus Cho/Cr for either responders (Res—light grey bars) or non-responders. **(C)** There are no between-group or within-group between-side (Ips vs. Cont) differences in thalamus NAA/Cr. Left vs. right interhemispheric % difference for Cho/Cr (**B**) or NAA/Cr (**D**) does not differ between responders (R) and non-responders (NR). Mann–Whitney tests were used to perform between-group comparisons, while Wilcoxon sign-rank tests were used to compare thalamus volumes within-groups. Statistical significance (*p* < 0.05) is indicated with *. Medians with interquartile ranges are displayed

There were no differences between pre-operative ipsilateral and contralateral VPM thalamus Cho/Cr in responders or non-responders. However, pre-operative ipsilateral Cho/Cr in non-responders (0.88, 0.78–1.06 IQR) was reduced compared to HCs (1.02, 0.98–1.13 IQR; *p* = 0.038). There were no other between-group or within-group between-side thalamus Cho/Cr differences (Fig. 5A, 5B).

There were no differences between ipsilateral and contralateral VPM thalamus NAA/Cr in responders or non-responders, and no other between-group or within-group between-side thalamus NAA/Cr differences (Fig. 5C, 5D).

#### Post-operative metabolite change at 1-week

**Fig. 6:**
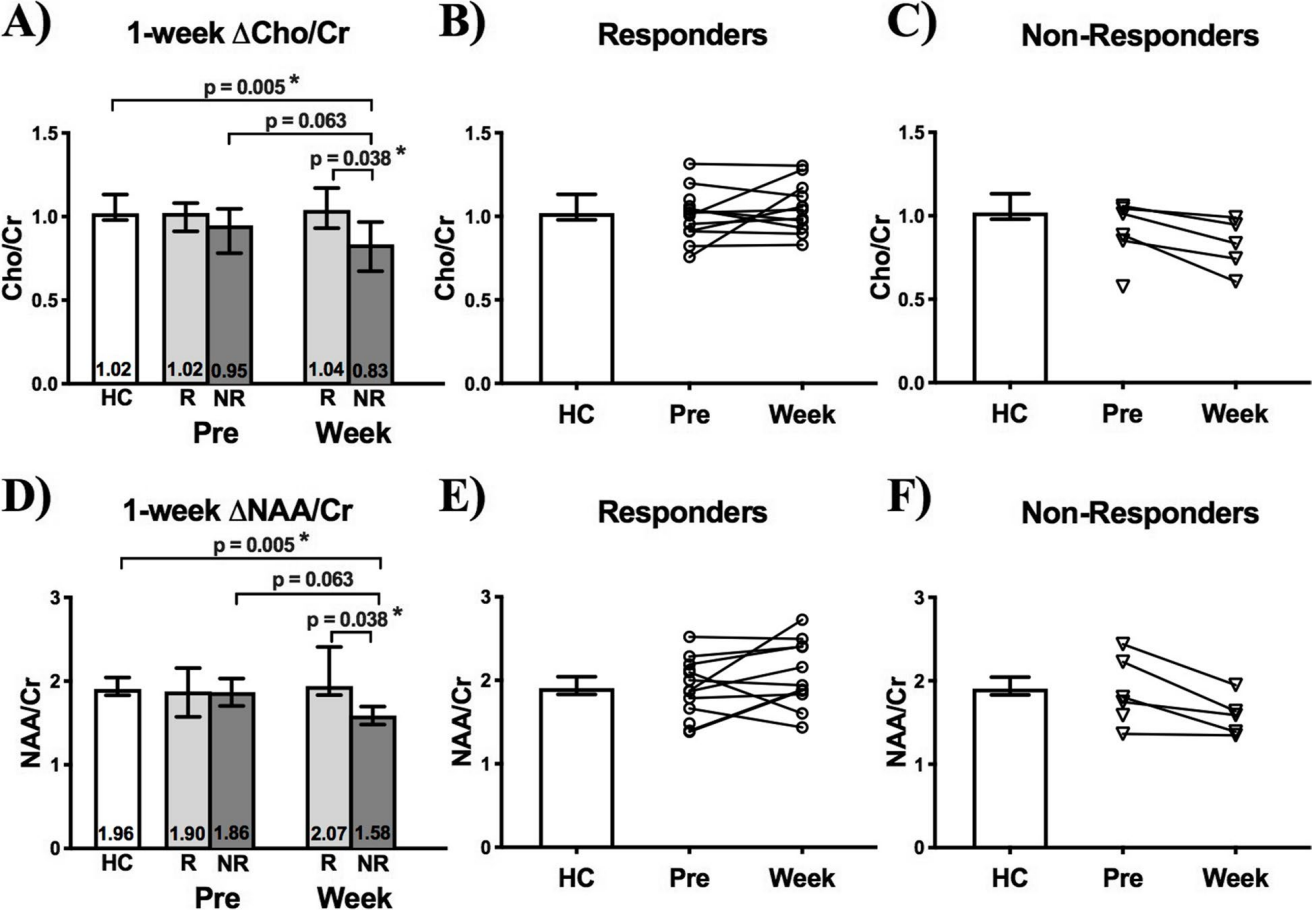
Peri-operative contralateral thalamus Cho/Cr and NAA/Cr change in TN patients 1-week following surgical treatment. **(A)** A strong trend suggests that Cho/Cr decreases in non-responders (NR—dark grey bars) 1-week after surgery (p = 0.063), at which point Cho/Cr differs (p = 0.038) from responders (R—light grey bars). **(B)** Cho/Cr either increases or decreases in responders at the individual-subject level, while **(C)** all non-responders show a decrease in Cho/Cr. **(D)** A strong trend suggests that NAA/Cr decreases in non-responders 1-week after surgery (p = 0.063), at which point NAA/Cr differs (p = 0.038) from responders. **(E)** NAA/Cr either increases or decreases in responders at the individual-subject level, while **(F)** all non-responders show a decrease in NAA/Cr. Mann–Whitney tests were used to perform between-group comparisons, while Wilcoxon sign-rank tests were used to compare thalamus volumes within-groups. Statistical significance (p < 0.05) is indicated with *. Medians with interquartile ranges are displayed

Pre-operatively, average VPM thalamus Cho/Cr in HCs did not differ compared to pre-operative contralateral Cho/Cr in either responders or non-responders (Fig. 6A). Furthermore, pre-operative contralateral Cho/Cr did not differ between responders and non-responders (*p* = 0.32). One week after surgery, Cho/Cr in non-responders was significantly lower than in responders (0.83, 0.67–0.97 IQR and 1.04, 0.93–1.17 IQR respectively; *p* = 0.038) and HCs (p = 0.005), and every non-responder for whom data was available showed a reduction in Cho/Cr irrespective of surgical procedure type (n = 3 MVD and n = 2 BC). Conversely, responders showed no Cho/Cr change with surgery (*p* = 0.57). (Fig. 6A-C).

Mirroring Cho/Cr, pre-operative average VPM thalamus NAA/Cr in HCs did not differ compared to pre-operative contralateral VPM thalamus NAA/Cr in responders or non-responders (Fig. 6D). Furthermore, pre-operative contralateral VPM thalamus NAA/Cr did not differ between responders and non-responders (p = 0.77). One week after surgery, NAA/Cr in non-responders was significantly lower than in responders (1.94, 1.83–2.41 IQR and 1.59, 1.37–1.79 IQR respectively; *p* = 0.038) and HCs (*p* = 0.005), and every non-responder for whom data was available showed a reduction in NAA/Cr irrespective of surgical procedure type (n = 3 MVD and n = 2 BC). Conversely, responders showed no NAA/Cr change with surgery (*p* = 0.20). (Fig. 6D-F).

## Discussion

In this single-centre, prospective, longitudinal study, we identified several abnormalities of thalamic structure and metabolism in medically-refractory TN patients undergoing surgery. Pre-operatively, TN patients showed increased thalamus volume contralateral to the painful side of the face, confirming earlier retrospective findings [22]. Shape analysis showed characteristic areas of the contralateral thalamus that were larger in responders to surgery compared to non-responders who experienced early pain recurrence. Metabolically, right-sided TN patients showed reduced NAA/Cr concentration in the contralateral left VPM thalamus, but this was not observed for left-sided TN patients. Non-responders to surgery showed significantly reduced pre-operative ipsilateral VPM thalamus Cho/Cr. Following surgery, we found novel evidence of metabolic changes in the VPM thalamus occurring as early as one week after either MVD or BC. These changes—specifically a significant reduction in Cho/Cr and NAA/Cr—were observed only in non-responders, suggesting that surgery for TN has variable effects on thalamic metabolism which may impact long-term pain outcome.

Overall, our patients demonstrated a 74% surgical response rate, in agreement with prior literature, notwithstanding differences in how surgical outcome is measured between different studies [3]. All TN patients were taking antiepileptic medication, and while medication use was largely the same between responders and non-responders, a greater proportion of non-responders were also taking baclofen, perhaps reflecting more exhaustive attempts at medical management. In line with previous reports, non-responders were more likely to be female, younger at the time of surgery, and had TN for nearly twice as long as responders [31]. NVC was identified on pre-operative MRI in 17 of 23 TN patients. At a group-wide level, there was no significant difference in NVC severity between responders and non-responders. However, it is worth noting that 50% of non-responders (3/6) but only 18% of responders (3/17) had no evidence of arterial NVC (i.e., NVC score 0), which may represent a relevant if not statistically significant difference. On reviewing these particular patients in greater detail, there were no obvious clinical or surgical factors that could explain response other than that all 3 non-responders with NVC score 0 were female. Interestingly, patient #23 was the only non-responder with NVC score 3 (i.e., distortion); again, this patient was female, and experienced pain recurrence within one year despite technically successful (i.e., confirmed on post-operative imaging) MVD. Taken together, our data are consistent with the notion that some female patients with a diagnosis of TN—whether in association with true NVC or not—may have a particularly treatment-resistant manifestation of the disease, perhaps explained by a distinct, though as yet unknown, pathophysiology [32].

Despite the established role of the thalamus in chronic and neuropathic pain [17], and as a component of the trigeminal sensory system, there have been few in-depth investigations of thalamic structure in TN [18, 19]. We found no whole-thalamus volume differences between HCs and TN patients, aligning with the previous work of Gustin et al*.* [19]. We did observe left–right volume asymmetry in HCs, which has been observed with many other subcortical structures (e.g., hippocampus), and may reflect normal functional and structural lateralization in the healthy brain. In line with our previous retrospective report [22], we also observed that the contralateral thalamus was larger across all TN patients, irrespective of the side-of-pain. Accordingly, it would appear that relative side (i.e., ipsilateral/contralateral) is the primary determinant of structural thalamic asymmetry in TN patients, possibly reflecting thalamic enlargement that is the result of sustained hyperactivity in the trigeminal system, similar to enlargement that has been observed in other chronic pain conditions [33, 34]. These data also confirm that the trigeminal system is affected at least as far upstream as the nuclei of third-order neurons residing in the contralateral thalamus. Prior to surgery, our data did not show clear evidence of differences in whole-thalamus volume between responders and non-responders, though more detailed vertex-wise shape analysis revealed a significant, circumferential band of contralateral thalamic volume loss in non-responders compared to responders. Given the small number of non-responders (n = 6), our study was likely underpowered to show statistically significant reductions in whole thalamus volume. However, the robust shape differences we observed suggest that non-responders have a structurally different contralateral thalamus which may play a role in conferring treatment-resistance. Given a reasonable degree of spatial overlap of this circumferential band with the VPM and medial dorsal nuclei, we speculate that volume loss may represent a form of trigeminal system injury—perhaps the result of prolonged chronic TN pain—which is more noticeable in non-responders, in whom it potentially dampens the effect of surgical interventions carried out at more peripheral locations (i.e., at the level of CNV). However, the lack of any significant difference in pre-operative pain severity (measured using the VAS) between responders and non-responders, would suggest that focal thalamic volume loss of this nature does not seem to alter pain relay function in the trigeminal system per se.

Unlike thalamic volume, we did not observe robust evidence of inter-hemipsheric asymmetry in metabolite concentrations of either Cho/Cr or NAA/Cr in the VPM thalamus in HC or TN patients pre-operatively. A trend suggests that NAA/Cr—an indicator of neuronal viability and number [35]—may be elevated in the left thalamus of HCs (p = 0.060), and may be further evidence of normal asymmetry between the left and right thalamus in humans. We did find that NAA/Cr was reduced in the contralateral left VPM thalamus in right-sided TN patients, perhaps reflecting reduced thalamic neuronal integrity that has been shown to be associated with the chronic pain state [21]. However, we did not observe this in left-sided TN patients, perhaps due to the smaller number of LTN patients in our cohort (n = 8). In line with our results, Wang et al*.*also found reductions in “posterior medial” and “posterior lateral” contralateral thalamus NAA/Cr in TN patients versus HC, and similarly found no difference in Cho/Cr—a marker of membrane turnover which if elevated may indicate a heightened state of cellular proliferation or inflammation—between these groups [21, 36]. Of note, Wang et al*.* did not limit their analysis to the VPM thalamus, using instead a multi-voxel MRS approach to examine several different thalamic subdivisions, though there is considerable spatial overlap between the VPM as we defined it and their “posterior medial” thalamus.

We found novel metabolic alterations in the thalamus which appear to be implicated in durable response to surgery for TN. First, we observed that non-responders had significantly lower pre-operative Cho/Cr concentration compared to HCs in the VPM thalamus ipsilateral to the side-of-pain. At first glance, this finding seems counterintuitive, since the bulk of trigeminal afferents to the VPM carry sensory information from the contralateral side of the face. That being said, there is neuroanatomical evidence that the smaller, ipsilateral dorsal trigeminothalamic tract containing non-decussating fibers may be implicated in orofacial pain [37], and recent in vivo tractography results show ipsilateral thalamo-cortical diffusivity abnormalities in TN [18]. Our current observations add to these findings, and further link surgical response to bilateral, system-wide changes in the trigeminal system.

Our most interesting finding is that of very early (1-week) metabolic changes occurring following TN surgery. While thalamus volume remained constant over this time interval, both contralateral Cho/Cr and NAA/Cr showed divergent changes between responders and non-responders: all non-responders had 1-week decreases in both metabolites, while responders maintained stable concentrations post-operatively. We speculate that these findings further suggest—in conjunction with shape analysis results—that there exists a degree of injury to the contralateral thalamus in patients with highly refractory and surgically resistant TN, which prevents it from responding appropriately to upstream surgical interventions.

Our study is limited by a relatively small sample size of 23 TN patients (19 in MRS analysis) and 20 HCs, and our findings will need to be replicated in larger TN datasets. However, our sample size is comparable to other prospective MRI-based studies of the thalamus in TN [18, 19, 21]. Another possible limitation is the heterogeneity of the TN group, which included patients who underwent different types of surgical procedures (MVD and BC), and in some cases repeat surgery. That being said, we did not observe any difference in thalamic volume or metabolite concentrations between patients treated with different surgical approaches, or undergoing non-virgin surgical treatment, suggesting that the central role of the thalamus in treatment responsiveness—or resistance—may be independent of interventions carried out more peripherally in the trigeminal system. Furthermore, while group-level medication-class use was largely the same between responders and non-responders, we did not have access to data on exact medication dosage and duration of use—both of which could have influenced the thalamic structural and metabolic abnormalities we observed—and could not correct for these in our statistical analyses. Finally, we arbitrarily defined responders as patients who continued to have satisfactory pain relief at one year following surgery, though it is certainly possible that some of these patients could have gone on to have significant pain recurrence at more delayed follow-up.

## Conclusions

Our findings confirm that trigeminal system abnormalities exist in TN patients as far upstream as the thalamus, evidenced by structural and metabolic alterations primarily—though not exclusively—in the thalamus contralateral to the side-of-pain. TN patients who respond inadequately to surgery exhibit baseline differences in thalamic shape, and differing trajectories of early post-operative change in thalamic metabolism, compared to responders. We conclude that the thalamus has a critical role to play in the pathophysiology of TN and its response to surgical treatment. The clinical implications of this finding at the individual subject level are an important topic for further research.

## Supplementary Information

**Additional file 1:** Supplementary** Figure S1**: 1H-MRS chemical shift image (CSI) slab placement and ventral posteromedial (VPM) thalamus voxel selection. CSI slab placement is shown in mid-sagittal (A), coronal (B), and axial (C) views, overlaid on T1-weighted MPRAGE images. Bilateral VPM thalamus voxel (indicated in yellow) is shown in coronal (B) and axial (C) views.

## Data Availability

Data available on request due to privacy/ethical restrictions. Please contact corresponding author Dr. Tejas Sankar (tejas.sankar@gmail.com) to request data.

## Abbreviations

TN: Trigeminal neuralgia
HC: Healthy control
CNV: Trigeminal nerve (i.e., cranial nerve V)
REZ: Root entry zone;
MVD: Microvascular decompression
MRI: Magnetic resonance imaging
DTI: Diffusion tensor imaging
VPM: Ventral posteromedial thalamus
MPRAGE: Magnetization-prepared rapid acquisition gradient echo
MRS: ^1^H-magnetic resonance spectroscopy
PRESS: Point-resolved spectroscopy
BC: Balloon compression rhizotomy
BNI: Barrow neurological institute
NAA: N-acetylaspartate
Cho: Choline
Cr: Creatine
VAS: Visual analogue pain score
IQR: Inter-quartile range
NVC: Neurovascular compression
IN: Internal neurolysis
