# Supplementary figures and images for: The thalamus in trigeminal neuralgia: structural and metabolic abnormalities, and influence on surgical response
Source: BMC Neurol. 2021 Jul 24;21:290. doi: 10.1186/s12883-021-02323-4 (PMC8305513; doi:10.1186/s12883-021-02323-4)

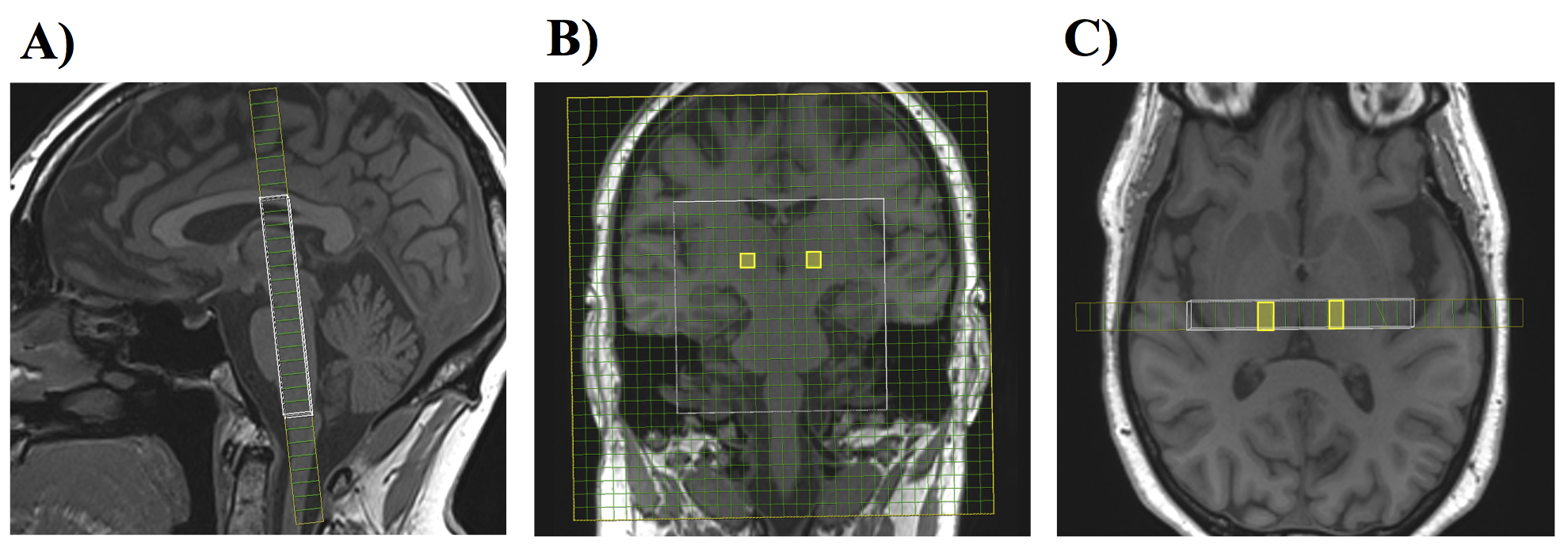

Supplement: Supplementary file 1 — Additional file 1: Supplementary Figure S1: 1H-MRS chemical shift image (CSI) slab placement and ventral posteromedial (VPM) thalamus voxel selection. CSI slab placement is shown in mid-sagittal (A), coronal (B), and axial (C) views, overlaid on T1-weighted MPRAGE images. Bilateral VPM thalamus voxel (indicated in yellow) is shown in coronal (B) and axial (C) views. [file 12883_2021_2323_MOESM1_ESM.tiff]
